# Patient Advocacy Assessment in the Medicine Clerkship: A Qualitative Study of Definition, Context, and Impact

**DOI:** 10.1007/s11606-021-07359-3

**Published:** 2022-02-07

**Authors:** Elizabeth P. Griffiths, Cindy J. Lai, Tali Ziv, Deanna Dawson, Gurpreet Dhaliwal, Margaret Wheeler, Arianne Teherani

**Affiliations:** 1 Department of Medicine, University of California, San Francisco, San Francisco, CA USA; 2 Department of Medicine, Kaiser Permanente, Oakland, CA USA; 3 Department of Obstetrics and Gynecology, Louisiana State University, New Orleans, LA USA; 4 Department of Medicine, San Francisco Veterans Affairs Medical Center, San Francisco, CA USA; 5 Department of Medicine, Zuckerberg San Francisco General Hospital, San Francisco, CA USA

**Keywords:** Advocacy, Assessment, Competency, Undergraduate medical education, Social determinants of health

## Abstract

**Background:**

Advocacy is a core value of the medical profession. However, patient advocacy (advocacy) is not uniformly assessed and there are no studies of the behaviors clinical supervisors consider when assessing advocacy.

**Objective:**

To explore how medical students and supervisors characterize advocacy during an internal medicine clerkship, how assessment of advocacy impacted students and supervisors, and elements that support effective implementation of advocacy assessment.

**Design:**

A constructivist qualitative paradigm was used to understand advocacy assessment from the perspectives of students and supervisors.

**Participants:**

Medical students who completed the internal medicine clerkship at UCSF during the 2018 and 2019 academic years and supervisors who evaluated students during this period.

**Approach:**

Supervisor comments from an advocacy assessment item in the medicine clerkship and transcripts of focus groups were used to explore which behaviors students and supervisors deem to be advocacy. Separate focus groups with both students and supervisors examined the impact that advocacy assessment had on students’ and supervisors’ perceptions of advocacy and what additional context was necessary to effectively implement advocacy assessment.

**Key Results:**

Students and supervisors define advocacy as identifying and addressing social determinants of health, recognizing and addressing patient wishes and concerns, navigating the health care system, conducting appropriate evaluation and treatment, and creating exceptional therapeutic alliances. Effective implementation of advocacy assessment requires the creation of non-hierarchical team environments, supervisor role modeling, and pairing assessment with teaching of advocacy skills. Inclusion of advocacy assessment reflects and dictates institutional priorities, shapes professional identity formation, and enhances advocacy skill development for students and their supervisors.

**Conclusions:**

Students and supervisors consider advocacy to be a variety of behaviors beyond identifying and addressing social determinants of health. Effectively implementing advocacy assessment shapes students’ professional identity formation, underscoring the critical importance of formally focusing on this competency in the health professions education.

**Supplementary Information:**

The online version contains supplementary material available at 10.1007/s11606-021-07359-3.

## INTRODUCTION

Advocacy on behalf of patients is a core value of the medical profession.^1,2^ However, advocacy is not considered a core competency in US medical schools, and how to define, teach, and assess this skill remains challenging.^3^ Defining and assessing patient advocacy is critical in the formative clerkship year when students often advocate for their patients while actively establishing their professional identities.^4^

Much of what is known about assessing health advocacy stems from countries that employ the CanMEDS framework, which includes serving as a health advocate among the core physician roles such as medical expert and communicator.^5^ For example, the University of British Columbia (UBC) Health Advocacy Framework defines advocacy as agency (helping patients navigate the health system) and activism (changing the underlying system).^6^ This definition also distinguishes between shared advocacy, in which patients and communities define needs, and directed advocacy, in which physicians use a biomedical lens to determine health needs.

Despite these frameworks, both learners and clinical supervisors describe the health advocate role as a challenging competency to assess partly because of lack of consensus about definition and included behaviors.^7^ Learners struggle to define its concrete behaviors and instead rely upon a sense of going “above and beyond” to define the role.^8^ There are no studies of the behaviors supervisors consider when assessing patient advocacy.

In this study, we explored how medical students and supervisors (residents and attendings) characterize patient advocacy during an internal medicine clerkship. We also examined the impact that assessment had on students’ and supervisors’ perceptions of advocacy and elements that support effective implementation of advocacy assessment.

## METHODS

### Design

We used a constructivist qualitative paradigm to explore how supervisors and medical students conceptualized patient advocacy and to determine how assessment impacted perceptions of advocacy.^9^ Our study was conducted during two academic years: 2018 and 2019. This study was approved by the institutional review board for all methods of data collection at the University of California, San Francisco (UCSF).

### Setting and Context

Our study was conducted at a public medical school in the western United States. Internal medicine is one of seven core clinical clerkships of the third year of medical school. Approximately 138 (81%) of students in the core clerkship year attend an inpatient 8-week block clerkship, while the remaining 32 (19%) complete their medicine clerkship in a longitudinal integrated clerkship (LIC). This study focused on advocacy assessment in the block (not LIC) version of the medicine clerkship as that is the most prevalent model for clinical education at our institution and nationally.^10^ Most students have new faculty and resident supervisors approximately every 2 weeks; supervisors must work with a student for at least 7 days to evaluate them.

In January 2018, the medicine clerkship implemented an advocacy item in students’ clerkship evaluation by supervisors to improve the comprehensiveness and equity in assessment. In our earlier work, we describe the change process that prompted the advocacy assessment pilot.^11^ The medicine clerkship was studied given the clerkship leadership’s interest in piloting this assessment and the potential to inform incorporation by other clerkships in the future.

### Instruments, Participants, and Sampling

We used written supervisor comments from the patient advocacy assessment item in the medicine clerkship student evaluation form and transcripts from focus groups to explore which behaviors students and supervisors deem to be advocacy. Separate focus groups with both students and supervisors were used to characterize the impact of advocacy assessment on students’ and supervisors’ perceptions of advocacy and what additional context was necessary to effectively implement advocacy assessment.

#### Patient Advocacy Assessment Item

In the patient advocacy assessment item, supervisors rated a “student’s advocacy in direct patient care activities.” Anchor ratings from 1 to 4 demarcated different levels of ability to identify and address “sociocultural factors that impact patient care, including (but not limited to) race, religion, culture, gender identity, sexuality, primary language, immigration status, and disability (ability).” The full text of the anchor ratings and a supplemental explanatory document provided to supervisors is available in Appendix 1. A free-text response field was also provided for open-ended written narrative comments to be submitted electronically on students’ advocacy skills. In 2018, the patient advocacy assessment item scores and comments were not included in final grades; in 2019, they were included. Students had access to all supervisor comments in all assessment domains after completion of the clerkship.

We stratified the narrative item comments based on three tiers (low, middle, high) of mean patient advocacy assessment scores. Ten students’ narrative assessment comments from each tier of performance from each year were chosen using a random number generator. These were reviewed to ensure a balance of student demographic characteristics (gender, under-represented in medicine status, and clerkship site). Substitutions based on demographics were made to create a final balanced set of sixty students’ evaluations (thirty from each year) to analyze.

#### Focus Groups

We conducted three student and two supervisor focus groups in 2019 with students and supervisors from the medicine clerkship during the 2018 and 2019 academic years. Students were invited to participate in the focus groups if they had completed the medicine clerkship within the prior or current year, or were in the final week of the clerkship. Supervisors were invited if they had evaluated students at least once since the introduction of the patient advocacy assessment. Students and supervisors were invited by email up to three times.

Two investigators (EPG, TZ) developed structured focus group guides (Appendix 2) to understand the participants’ definitions of patient advocacy, behaviors they considered advocacy, and whether the addition of the advocacy assessment led to the provision of formative feedback, signaled the importance of physician advocacy, and/or changed students’ or supervisors’ behavior in caring for patients. Participants were also queried on ways to improve advocacy assessment and instruction. An investigator who was not a clerkship director or involved in the clerkship assessment process (EPG) conducted all focus groups.

### Data Analysis

We used a thematic analysis approach to analyze supervisor comments about students’ patient advocacy and supervisor and student focus group data.^12^

#### Patient Advocacy Assessment Item

Four investigators (EPG, CL, GD, MW) reviewed the supervisor comments for 30 students to develop a codebook. After discussion, EPG revised the codebook. Comments for 20 students were then double coded (EPG and GD, CL and MW) using the revised codebook. Areas of disagreement were reconciled, and the codebook was finalized. The final codebook was then applied to the remaining 40 students’ supervisor comments by individual investigators. When areas of ambiguity arose, investigators conferred until consensus was achieved.

#### Focus Groups

All focus groups were recorded and transcribed. Four investigators (EPG, CL, TZ, AT) reviewed one supervisor and one student focus group transcript to develop an initial codebook and then met to refine codes. The revised codebook was then applied to a different transcript by all four investigators. After group discussion, the codebook was finalized. The remainder of the transcripts were coded by two investigators with differences reconciled prior to finalizing coding. We used Dedoose software (SocioCultural Research Consultants, LLC version 7.5.9) for coding.

### Researcher Reflexivity

The investigator team consisted of one clinician educator involved in teaching advocacy in other parts of the medical school curriculum (EPG), one fourth year medical student (DD), one medicine clerkship director (CL), three medicine clerkship site directors (TZ, GD, MW), and one education researcher (AT). The diverse background of the investigators allowed for varying insight throughout all phases of the study.

## RESULTS

Supervisor comments for the patient advocacy assessment item for 60 students from the three tiers of performance (30 for 2018, 30 for 2019) were analyzed. Two supervisor focus groups with 16 supervisors in total and three focus groups with 13 students in total were conducted. To develop a rich conceptualization of patient advocacy, we present our results from the analysis of student and supervisor focus groups integrated with patient advocacy assessment item narrative comments.

### Patient Advocacy Definition and Behaviors

We describe behaviors that participants defined as advocacy with example quotes provided in Table 1.

**Table 1:** Patient Advocacy Behaviors

**Advocacy behavior**	**Representative quotes**
Recognizing and addressing social determinants of health	• “[She] deftly incorporated patients' sociocultural status into her care and their treatment plans…she managed an elderly frail gentleman with multiple heart failure exacerbations with exceptional care: she did extensive discharge counseling, incorporated his brother/caretaker and ensured he had adequate support at home.” (S2018-19) • “[He] is a phenomenal advocate for his patients. One of his patients was a young gentleman who had been hospitalized for months. [He] quickly caught on to this young man's psychoemotional distress and learned that the patient was a devout Catholic. [He] went out of his way to make sure that a Catholic priest could visit the patient on a regular basis” (S2018-24)
Navigating the complex health care system	• “She advocated for his care in the acute setting but also worked to link all of his providers (mental health, nurses, PCPs, case managers, social workers) into his care while an inpatient recognizing that for this particular patient he needed a fully functioning support system.” (S2018-20)
Recognizing and addressing patient concerns and goals	• “[He] was an excellent advocate for his patients and prioritizes providing care in a patient-centered manner. In one instance his patient request[ed] to leave the hospital for several hours for a graduation. Instead of labelling this as an "AMA" (against medical advice) [he] worked tremendously hard to coordinate a smooth transition out and back into the hospital.” (S2019-28) • **“**[He] always prioritized his patients concerns during his presentations making sure his patients' concerns were heard.” (S2019-16)
Advocating for appropriate medical evaluation and treatment	• “We had an undocumented patient who was newly diagnosed with cancer and [he] made sure to advocate for expediting the patient's care while in-house.” (S2018-4)
Exceptional therapeutic alliance and addressing mistrust	• “She always KNEW her patients. She knew them beyond their presenting complaint or their medical history. She know who they were, what they valued, and how we could tailor care in order to best serve them.” (S2018-25) • “[She] advocated for this patient to our entire team and pushed us to consider the historical and social context of this gentleman's current hospitalization to understand his apprehension in trusting his medical team. It was through her care that we were able to slowly build a positive relationship of trust with this gentleman.” (S2019-23)

Each student has been labeled with a participant ID beginning with S followed by their year (2018 or 2019) and their participant number (1–30)

#### Recognizing and Addressing Social Determinants of Health

Recognizing and addressing social determinants of health was identified by all participants as advocacy. This could take many forms, including collaboration with interpreters, spending time educating patients and families with low health literacy, or collaborating with the interdisciplinary team to address financial barriers to care.

#### Navigating the Complex Health Care System

Participants described helping patients navigate the fragmented health care system or mitigating its inequities as advocacy. At the individual patient-level, this included care coordination, obtaining medical records, ensuring patients would receive medications and supplies upon discharge, scheduling follow-up appointments, arranging transportation, and providing continuity by following the patient longitudinally. At a systems-level, this form of advocacy included working to address inequities perpetrated by the health care system and “pushing back” against insurance company and hospital policies that were not in the patient’s best interest.

#### Recognizing and Addressing Patient Concerns and Goals

Advocacy behaviors included listening to and eliciting patient goals and concerns, communicating these to the medical and interdisciplinary team, and aligning care plans with patient values. Supervisors particularly noted when students worked with patients with limited English proficiency or from marginalized groups or were willing to disagree with the team’s plan in order to address patients’ goals.

#### Advocating for Appropriate Medical Evaluation and Treatment

Ensuring patients receive the appropriate medical evaluation and consultation with specialists was considered patient advocacy. This was a point of debate and ambiguity among both supervisors and students. Some argued that advocating for appropriate medical evaluation was too broad a definition of advocacy. Others thought that exceptional efforts at ensuring patients received appropriate medical evaluation and treatment is advocacy, particularly when supporting patients from marginalized groups or when addressing underlying mental health conditions, substance use disorders, chronic pain, and untreated symptoms.

#### Exceptional Therapeutic Alliance and Addressing Mistrust

Spending the time to get to know patients as people beyond their medical conditions or hospital stay and incorporating this knowledge into care plans was seen as advocacy. Examples included involving patients’ families or trusted community members in care; demonstrating respect, compassion, or empathy for marginalized patients; and demonstrating knowledge of the patient with small gestures that acknowledged their humanity, such as bringing a favorite food or celebrating a birthday. Recognizing patients’ mistrust of the health care system was viewed as especially important.

There were no differences in the types of advocacy behaviors evaluators commented on between students who scored in the low, middle, and high tiers on patient advocacy. However, students in the latter group received more comments citing specific stories and behaviors. Lower scoring students were more likely to receive vaguely positive comments lacking details, often referring to their general awareness of social determinants of health but not how they translated this into specific actions to help their patients.

### Essential Context for Patient Advocacy Assessment

Participants described context essential to implement fair and accurate advocacy assessment. Themes are outlined below with representative quotes provided in Table 2.

**Table 2:** Essential Context for Effectively Implementing Patient Advocacy Assessment

**Essential context**	**Representative quotes**
Supervisors should create positive, non-hierarchical team environments that encourage and acknowledge patient advocacy.	“The only times I've been nervous is just I think it's contradicting other team members… I don't think I would've ever done that if I didn't feel so comfortable. My attendings and my residents had really empowered me.” (SFG 1)^1^
Supervisors should role model patient advocacy.	“Going back to this same patient I was talking about looking for his wheelchair, my intern and I, we didn't have access to all these places but our attending actually walked down with us to look for it. I think that was another example of patient advocacy.” (SFG 3)
Supervisors should provide frequent, specific feedback on patient advocacy behaviors based on direct observation.	“If it's just the one [evaluation] at the end, by the time that they realize this, it's over. So, it doesn't really help you… having it built in to some form of… midway feedback thing where you have to go through this together or some kind of quick education on it at the beginning before the new rotations are going to start.” (SFG 1)
Clerkship leadership should disseminate clear guidelines defining patient advocacy, including example behaviors and how to score and comment on patient advocacy.	“It would be helpful to have a framework …like what a reporter is for advocacy, what an interpreter is, what is a manager, what is an educator?” (CSFG 1)^2^
Clerkship leadership should develop and expand complementary teaching of patient advocacy skills.	“I think there are other ways to encourage people to be advocates for their patients. One of the ways is to say, we're going to start assessing you on this… versus having that become more of an explicit part of the curriculum going forward.” (CSFG 1)

^1^*SFG*, student focus group. Each student focus group has been labeled as 1, 2, or 3

^2^*CSFG*, clinical supervisor focus group. Each clinical supervisor focus group has been labeled as 1 or 2

#### Supervisors Should Create Positive, Non-hierarchical Team Environments That Encourage and Acknowledge Patient Advocacy

Students and supervisors reported that encouraging students to share their opinions, even when different from their supervisors, empowered students to advocate for their patients. Students appreciated when teams elicited student input and provided reinforcing feedback. They noted that it was challenging to advocate when the team dynamic felt hierarchical or senior members of the team did not practice or value advocacy. Some students worried that team environment variability would be a challenge to fair advocacy assessment, but students hoped that assessment would promote a change in team culture.

#### Supervisors Should Role Model Patient Advocacy

Both students and supervisors remarked on the importance of supervisors modeling advocacy in order for students to succeed at this role. Students praised supervisors who role modeled respect for patients, including talking with patients at eye-level, asking open-ended questions, and obtaining a detailed social history. Students appreciated learning how supervisors helped patients navigate the system in ways beyond their expected skills as medical students, including providing detailed knowledge about resources, advocating for resources at multidisciplinary rounds, and talking with administrators and insurance companies when a decision was being made against the patient’s best interests. Supervisors agreed that their role modeling was essential although expressed concerns that system constraints might prevent them from ideal modeling.

#### Supervisors Should Provide Frequent, Specific Feedback on Patient Advocacy Behaviors Based on Direct Observation

Students and supervisors suggested that all team members should become familiar with the patient advocacy assessment item early in the rotation. Formative feedback on advocacy skills throughout the rotation was more useful than summative feedback at the end. Both students and supervisors noted the challenge of assessing student advocacy when faculty did not observe students’ acts of advocacy for patients. Residents sometimes filled this direct observation gap for faculty.

#### Clerkship Leadership Should Disseminate Clear Guidelines Defining Patient Advocacy, Including Example Behaviors and How to Score and Comment on Patient Advocacy

Despite communication from the clerkship leadership, some students and supervisors learned about the assessment for the first time when completing or reviewing evaluations. Specific guidance, using multiple modes of communication, would educate supervisors on how to use the numeric scale, and exemplar narrative comments should be provided. Another recommendation was to eliminate numeric ratings for this assessment item and rely solely on narrative comments.

#### Clerkship Leadership Should Develop and Expand Complementary Teaching of Patient Advocacy Skills

Both students and supervisors recommended that patient advocacy assessment item be part of a broader program of teaching advocacy skills during the clerkship year. Some supervisors were concerned about assessing advocacy without having a complementary curriculum for students to learn and develop advocacy skills.

### Impact of Patient Advocacy Assessment

Participants described the impact of the advocacy assessment on students and supervisors as outlined below with representative quotes displayed in Table 3.

**Table 3:** Impact of Patient Advocacy Assessment

**Impact**	**Representative quotes**
Reflects and dictates priorities	• “It totally reflects the values of the institution and the direction that [university name] wants to go in, in terms of pioneering and modeling the future of education for health equities and health disparities” (CSFG 2)^2^ • “I can imagine that …when you have a little bit of free time you can either use an hour of your time to go and talk to your patients and get to know them better, or read up on something else. And so there's definitely an opportunity cost there, and …making that clear that the advocacy part weighs as much as medical knowledge could incentivize someone to go talk to their patient.” (SFG 1)^1^
Shapes professional identity formation	• “It is nice… that students have seen that this is a part of what it means to be a good doctor. It's not just the things that you do that are nice. It is what makes you good.” (CSFG 1)
Allows for skill development and specific feedback	• “It would give me a chance to reflect on what I'm doing right and what I should continue doing if I'm getting the feedback that I did a great job of patient advocacy for this. So I think it'll serve as a reminder for future patients.” (SFG 3)

^1^*SFG*, student focus group. Each student focus group has been labeled as 1, 2, or 3

^2^*CSFG*, clinical supervisor focus group. Each clinical supervisor focus group has been labeled as 1 or 2

#### Reflects and Dictates Priorities

Students and supervisors found that assessing advocacy marked it as a professional and institutional value. They speculated that assessment prompts reluctant supervisors or teams to prioritize advocacy.

Defining advocacy as a core competency elevated advocacy among competing demands. Many students felt that the advocacy assessment allowed recognition of an unacknowledged role often assumed by students. Some supervisors felt that students should not be expected to advocate for patients, but that the domain provided an opportunity to acknowledge students who go “above and beyond” for patients. No students shared this concern, and many expressed gratitude to be given “credit” for work aligned with their values. Supervisors also felt an increased pressure to advocate for patients when students are assessed on the skill.

Some students felt that assessment alone could not dictate a team’s priorities. They cited a culture that values feedback on knowledge and presentations more than advocacy, and the importance of individual team environments in teaching and prioritizing skills. Several supervisors noted that it would take more faculty development to change ingrained practice and teaching patterns.

#### Shapes Professional Identity Formation

Assessing advocacy signaled that advocacy is a core competency of physicians, not an optional skill, and reminded students of why they had pursued a medical career. Seeing supervisors model advocacy emphasized it as essential to physician identity.

For some supervisors, assessing advocacy changed their mental model of a highly skilled clerkship student and that of an accomplished attending physician. Others felt that although they already valued advocacy, the assessment allowed them to acknowledge it explicitly.

#### Promotes Skill Development and Feedback

Students and supervisors considered advocacy assessment valuable because it encouraged specific feedback to enhance these skills. Students were hopeful that advocacy assessment would foster reflection on how to be a better physician rather than lead to the strategic pursuit of advocacy for a higher grade.

## DISCUSSION

**Figure 1:**
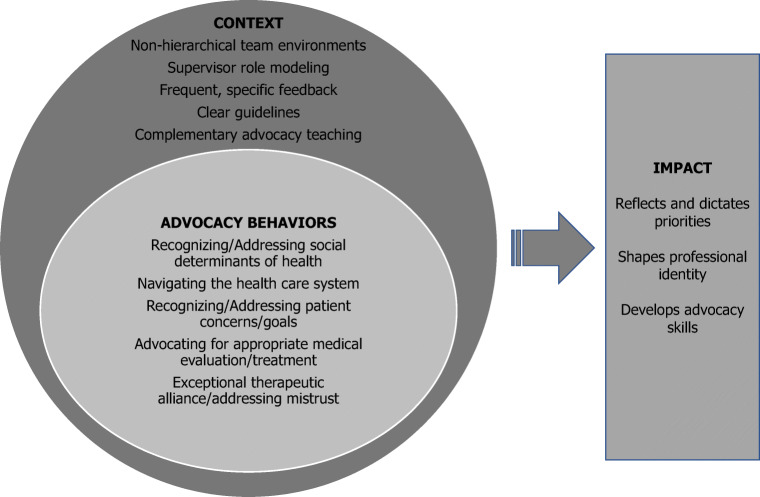
Context and impact of implementing effective patient advocacy assessment. The figure synthesizes the findings of our study. At the core of the figure, students and supervisors define patient advocacy through the behaviors they consider advocacy. Effective implementation of patient advocacy assessment, represented by the outer circle supporting the core in the figure, requires key contextual elements. And finally, inclusion of patient advocacy assessment impacts students and supervisors by reflecting and dictating institutional priorities, shaping professional identity formation, and enhancing advocacy skill development.

We sought to characterize how clerkship students and supervisors define patient advocacy, what context they consider necessary to effectively implement advocacy assessment, and the impact that advocacy assessment had on students and supervisors. We found that students and faculty define patient advocacy more broadly than clerkship leaders who conceptualized advocacy as identifying and addressing sociocultural factors that affect health. Hubinette and colleagues also demonstrated a lack of consensus among learners in definitions of health advocacy. They highlighted a tension between defining health advocacy as a set of behaviors versus a more general sense of going “above and beyond.”^8^ Navigating the health care system, facilitating access to resources, and expediting medical evaluation and treatment were behaviors identified as individual-level advocacy in both the Hubinette study and ours. The sense of going “above and beyond” may explain why evaluators did not report distinct behaviors for students who scored lower in this domain. In a separate study of patient advocacy assessment in the longitudinal integrated clerkships (LICs) at our institution, investigators also found that students and supervisors perceived developing strong therapeutic alliances and identifying and addressing patient needs, particularly for marginalized patients, as important patient advocacy behaviors.^13^ Based on our findings and those of others, we propose that patient advocacy be defined more broadly to include the behaviors outlined in Figure 1.

We found several factors that promote fair and accurate advocacy assessment. Many of these elements are common to any form of skill development and assessment, including frequent specific feedback based on direct observation and clear guidelines. These may be especially important for patient advocacy assessment given the ambiguity in defining the domain^6^ and lack of a standard advocacy skills-based curriculum.^14^ The importance of non-hierarchical team environments may be instrumental for students to excel in advocacy, which often requires challenging the status quo or other team members.

The impact of advocacy assessment on students and supervisors in our study underscores the power of assessment to drive priorities, shape professional identity, and develop specific skills. Complementary advocacy skills teaching sessions for students and faculty development—including defining and assessing patient advocacy, providing specific feedback regarding advocacy behaviors, and role modeling patient advocacy—are necessary to drive behavior change. As professional societies increasingly declare advocacy to be a core professional competency, accrediting bodies must consider how to integrate advocacy skills assessment throughout medical training. Broader training on advocacy for policy and systems change is also required to reduce the need for physicians to go “above and beyond” for their patients, which may contribute to burnout.

Much work remains to be done before we can fully describe best practices in teaching and assessing advocacy as a core competency in medical education. Future research should focus on defining advocacy behaviors and milestones at different levels of training, practice locations and specialties; how best to teach and assess advocacy skills at each level; and how to develop faculty and residents competent to do so.

This study has limitations. As a single-institution study, generalizability of findings may be limited. Our institution emphasizes social justice and advocacy as core objectives and may attract students and supervisors who are especially supportive of advocacy assessment. Furthermore, students and supervisors who choose to participate in focus groups may be more likely to support advocacy assessment.

In addition, we evaluated implementation of advocacy assessment in one clerkship (internal medicine) which may prompt different advocacy behaviors compared to other clerkships. The medicine clerkship was the first to implement patient advocacy assessment due to clerkship leadership interest, and the neurology and psychiatry clerkships are now incorporating advocacy assessment. Additional study in these new contexts will deepen our understanding of how patient advocacy may be defined similarly or differently in other specialties. We expect that the core categories of behaviors will likely be the same but that specific actions and their relative importance may differ across specialties.

Our study furthers understanding of which behaviors students and supervisors consider patient advocacy, broadening the definition from identifying and addressing social determinants of health to include recognizing and addressing patient wishes and concerns, navigating the health care system, conducting appropriate medical evaluation and treatment, and creating exceptional therapeutic alliances. Our findings that advocacy assessment shapes students’ professional identity formation, helps students to prioritize advocacy, and develops their advocacy skills reveal how critical it is that the health professions education community continues to formally focus on this competency. Effective implementation of advocacy assessment requires non-hierarchical team environments, supervisor role modeling, and teaching advocacy skills. If the medical profession wants to train physicians who have the skills to improve the health and well-being of their patients, their communities, and society, we must develop and assess the competencies that align with the values we profess.

## Supplementary information

ESM 1 (DOCX 22 kb)
