# Supplementary material for: Patient Advocacy Assessment in the Medicine Clerkship: A Qualitative Study of Definition, Context, and Impact
Source: J Gen Intern Med. 2022 Feb 7;37(10):2489–95. doi: 10.1007/s11606-021-07359-3 (PMC8821783; doi:10.1007/s11606-021-07359-3)
Supplement: Supplementary file 1 — (DOCX 22 kb) [file 11606_2021_7359_MOESM1_ESM.docx]

**Appendix 1. Focus Group Guides**

Introduction to student focus groups

Thank you for your willingness to participate in this focus group. In this focus group we hope to discuss your experiences with and perception of student advocacy on behalf of patients. We are also interested in your experience with being assessed around advocacy on behalf of patients and any impact on your learning environment. Your perception on this topic will be critical in improving future curricula and assessment of advocacy.

Background: In January 2018, with careful consideration by students, faculty, and leadership of the current vision and program objectives, as well as evidence on effective clinical teaching, a new section was added to the MedHub assessment of students in their F2 Medicine clerkship evaluations which focused on student advocacy for their patients. A rubric was created to support residents and faculty to evaluate students in this area and was distributed to all medicine clerkship faculty.

We will follow some guidelines as during this focus group. What you say will be strictly confidential and will be presented to the study working group (which includes your medicine clerkship leadership) only after your final grades are submitted. Your name and voice will **not** be associated with what you have said and we ask that the students at this group also keep what their classmates have said in confidence. To create an open and honest forum of ideas we ask that you respect each other’s ideas. We are eager to hear your personal experiences and would prefer that you focus on your experiences rather than those of classmates or peers. We will want to hear about the positives and areas for improvement. If you are unhappy with an experience, please provide suggestions for how those areas could have been designed or executed to better meet your learning needs.

Facilitator introduce themselves and their roles (e.g. might have assistant for administrative support). Our role as facilitators is to ensure we capture your perspectives, balance the conversation, and ensure the views of all participants in this group are captured and represented. We will use qualitative methods to analyze the data from this group which is being collected with the goal of assessing the intervention of adding a new domain of advocacy to assessments. This study may be published in a medical education journal and the information gathered may impact future curricula and assessment practices at UCSF.

Student focus group questions:

1. Let’s talk about assessment, meaning your evaluations in the medicine clerkship. Are you aware that there is a provisional domain of assessment on the Internal Medicine medical student assessment forms specific to patient advocacy that was added in January 2018?
   - 1. Probe: If so, how did you become aware of it?
        1. Probe: by email, did clerkship leadership tell you, through preceptor, or reading it on assessment /MedHub evaluation?
2. How do you define advocacy in the care of individual patients (otherwise known as patient-level advocacy)?
   - 1. Probe: I am clarifying that I am referring to advocacy by the medical team for the patient in the clinical context.
3. How do you see advocacy in your role as a future attending physician in the clinical context, caring for the individual patient?
4. How do you see advocacy fitting in or being part of your role as a future physician more generally?
5. Can you reflect on an example or examples of specific times you advocated for a patient during a clinical encounter on your medicine clerkship?
6. Did you notice your medicine clerkship attending advocate for individual patients in the clinical setting?
   - 1. Probe: Can you give an example?
     2. Probe: Did you notice other attendings advocating for individual patients in the clinical setting?
        1. Can you provide an example?
7. Did you notice your medicine clerkship attending miss a chance(s) to advocate for a patient in the clinical setting?
   - 1. Probe: Can you give an example?
     2. Probe: Did you notice other preceptors or attendings missing this chance?
        1. Can you provide an example?

At this time I am passing out the patient advocacy assessment item. Please take a moment to look over it and feel free to refer to it in the rest of this discussion.

1. Did your medicine attendings give you feedback about whether and/or how you advocate for your patients?
   - 1. Probe: Can you give examples?
     2. Probe: If you gave no examples, why might you not have advocated for a patient or not have received feedback on this skill?
2. How do you see your advocacy on behalf of the patient being recognized?
   - 1. Probe: For example, verbally? Written narrative? Scores? Can you expand?
3. When it does get recognized, how does that make you feel?
   - 1. Probe: Does it change the image you of have of the role of the physician to receive feedback around advocating for patients?
4. What are your thoughts about including patient advocacy among the other areas evaluated specifically, such as oral presentations, fund of knowledge, problem solving, system-based learning and communication/professionalism?
5. Do you feel this new section adds something useful to the other categories in which you are already evaluated?
   - 1. Probe: If so, can you elaborate?
6. Can you comment on whether assessing student advocacy for a patient in the medicine clerkship sends a message to you about the values of this medical school or this institution?
7. Do you think assessing a students’ advocacy skills changes student and team behavior?
   - 1. Probe: if so, how?
8. Do you think assessing student advocacy for a patient enhances equitable care?
   - 1. Probe: if so, how?
9. Does it allow or empower students to share their perspectives on barriers to care/ social vulnerability and increase the appreciation and regard for diverse perspectives?
   - 1. Probe: in what ways?
10. Does it decrease the tension students feel between advocating for the patients on the one hand and a sense that their advocacy might negatively influence their grade?
    - 1. Probe: can you provide examples?
11. Finally, what are your thoughts about including patient advocacy in evaluations going forward and why?
    1. Do you think the change in grading to remove honors will impact how students view inclusion of this assessment item?

Introduction to supervisor focus group

Thank you for your willingness to participate in this focus group. In this focus group we hope to discuss your experiences with and perception of student advocacy on behalf of patients. We are also interested in your experience with assessing students around advocacy on behalf of patients and other impacts of assessing student advocacy. Your perception on this topic will be critical in improving future curricula and assessment of advocacy.

Background: In January 2018, with careful consideration by students, faculty, and leadership of the current vision and program objectives, as well as evidence on effective clinical teaching, a new section was added to the MedHub assessment of students in their F2 Medicine clerkship evaluations which focused on student advocacy for their patients. A rubric was created to support residents and faculty to evaluate students in this area and was distributed to all medicine clerkship faculty.

We will follow some guidelines during this focus group. What you say will be strictly confidential and will be presented to the study working group (which includes clerkship leadership) only after final grades are submitted. Your name and voice will not be associated with what you have said and we ask that you keep what others shared in confidence within this group too. To create an open and honest forum of ideas we ask that you respect each other’s ideas. We are eager to hear your personal experiences and would prefer that you focus on your experiences rather than those of your peers. We will want to hear about the positives and areas for improvement. If you are unhappy with an experience, please provide suggestions for how those areas could have been designed or executed to better meet our students’ learning needs.

Facilitator introduce themselves and their roles (e.g. might have assistant for administrative support). Our role as facilitators is to ensure we capture your perspectives, balance the conversation, and ensure the views of all participants in this group are captured and represented. We will use qualitative methods to analyze the data from this group which is being collected with the goal of assessing the intervention of adding a new domain of advocacy to assessments. This study may be published in a medical education journal and the information gathered may impact future curricula and assessment practices at UCSF.

Supervisor focus group questions:

1. How do you define advocacy in the care of individual patients (otherwise known as patient-level advocacy)?
   - 1. What does patient advocacy in medicine mean to you?
2. Do you think patient advocacy has a role in medicine?
3. Do you feel that advocacy on behalf of the patient is relevant to your medical practice, and if so, how?
4. Are you aware there is a provisional domain of assessment on the internal medicine clerkship assessment forms that you complete specific to patient advocacy that was added in January 2018?
   - 1. Probe: If so, how did you become aware of it?
        1. Probe: Did you read an email, did clerkship leadership tell you, did a student tell you, or did you see it on the assessments you completed?
5. Can you reflect on an example or examples of specific times you advocated for a patient in the clinical setting recently?
6. Can you recall missing a chance(s) to advocate for a patient?
   - 1. Probe: Can you give an example?

How might you ideally model advocacy for your medical students in the clinical setting?

1. Has addition of the patient advocacy item impacted how you approach your own patient care?
   - 1. Probe: Why or why not?
     2. Probe: In what ways?
     3. Probe: Can you give an example?

Let’s talk about student assessment. For LIC: You complete formal written RIME assessments roughly quarterly, as well as BSCO informal observation feedback a few times a year, and you deliver informal verbal feedback regularly as well. Most if not all of you have precepted medical students prior to this year.

1. How do you recognize student advocacy on behalf of the patient?
   - 1. Probe: Do you talk with your students about it? Give verbal feedback? Written narrative?
2. What are ways you observe your students advocating for the patient in the clinical setting?
   - 1. Probe: What specific behaviors do you consider patient advocacy?
3. When you recognize student advocacy for a vulnerable patient verbally or on paper, have you considered whether this has changed your mental model of the highly skilled medical student?
   - 1. Probe: did it change your perception of the medical student identity?
4. Does the assessment of student advocacy of the patient change your own behavior in the clinical setting?
5. Does or would receiving positive feedback from a student for advocating for the patient change your perception of your identity as a physician?
   - 1. Probe: In what way does it change your perception of your identity?
6. Does including the section of patient advocacy among the other sections evaluated (such as medical knowledge, problem solving, oral presentations, and system-based learning) add anything novel to your assessment of students? If so what?
   - 1. Probe: Does space to comment on patient advocacy add a dimension to the student evaluation that wasn’t covered by prior existing domains of assessment?
     2. Probe: If it adds something, in what ways does it allow you to more accurately, holistically and fully evaluate the student’s action, behavior and skill overall?
     3. Probe: How is assessing patient advocacy different than assessing patient communication and team communication skills?
7. Does it send a message about the institution or the medical school that there is assessment of advocacy?
   - 1. Probe: If so, what message?
8. Do you think evaluating student advocacy for the patient changes the learning environment or student wellbeing?
   - 1. Probe: If so, in what ways?
9. Finally, what are your thoughts about including patient advocacy in evaluations going forward and why?

**Appendix 2. Patient Advocacy Assessment Item**

The patient advocacy assessment item asked clinical supervisors to rate “a student’s advocacy in direct patient care activities.” Anchor ratings from 1 to 4 were provided and range from:

1. Does not identify sociocultural factors that impact patient care, including (but not limited to) race, religion, culture, gender identity, sexuality, primary language, immigration status, and disability (ability) as important to patient care.
2. Can identify sociocultural factors that impact patient care, including (but not limited to) race, religion, culture, gender identity, sexuality, primary language, immigration status, and disability (ability) BUT requires prompting to analyze these factors in the context of an individual patient’s care.
3. Identifies sociocultural factors that impact patient care, including (but not limited to) race, religion, culture, gender identity, sexuality, primary language, immigration status, and disability (ability), AND is able to consider these factors in the context of an individual patient’s care, demonstrating understanding of the specific factors impacting that individual’s care.
4. Identifies sociocultural factors that impact patient care, including (but not limited to) race, religion, culture, gender identity, sexuality, primary language, immigration status, and disability (ability), AND incorporates these factors in the individual patient’s care AND implements solutions to overcome barriers.

A supplemental document further describing patient advocacy and potential examples was also provided for supervisors to link to. Its text is copied below:

Patient Advocacy Definition and Examples

The clerkship will focus primarily on a student’s advocacy in direct patient care activities. A student demonstrating a strong personal commitment to help his/her patient address the full spectrum of their needs with the goal to improve patient care, trust and experience and reduce health care disparities for vulnerable patients and/or populations. Specifically, the student who excels in this domain exhibits behaviors that would demonstrate that he/she consistently was able to incorporate patient factors including (but not limited to) race, culture, gender identity, sexuality, age, primary language, literacy, socioeconomic status, immigration status, illness status, self-efficacy, and disability (ability) into his/her care of the patient. The student utilizes his/her relationship with the patient and team to ensure maximal respect and equity for the patient. Conditions complicating medical care such as homelessness, histories of incarceration, substance use, mental illness or obesity are integrated sensitively into patient care plans. Students with advanced skills might extend their efforts by referring to outside resources, reviewing medical evidence and educating the team, collecting stories or considering using experiences to improve systems or advocate for policy changes to improve care more broadly.
Examples would include:
• proactively and appropriately engaging the use of interpreter services
• assisting patients with navigating the health care system
• using techniques/resources accounting for health literacy
• addressing a patient’s marginal housing status in discharge planning
• coordinating services with case-management or methadone treatment facilities
• advocating for patients with outside agencies: immigrations, work places; housing facilities,
parole boards, etc.
• facilitating communication between teams and patients/patient families and outside agencies or
resources around challenging or equity-related issues
• thinking creatively and identifying resources to ensure appropriate and equitable care for
patients with socioeconomic issues affecting their diseases (i.e., when the patient cannot afford
a medication)
• emphasizing the use of inclusive, respectful language in communication with team and
interdisciplinary colleagues
• seeking to understand the patient’s perspective of their disease which may help identify
additional care barriers or supports
• identifying resources to address an identified health disparity
• advocating while recognizing the limits of his/her expertise.
• seeking guidance from consultants, medical literature, other experts on best practices for social
vulnerabilities
